# Gynecological Cancers Among American Indian and Alaska Native Women Living in the Upper Midwest, 1995–2019

**DOI:** 10.1089/whr.2024.0124

**Published:** 2025-02-25

**Authors:** Keely K. Ulmer, Breanna Greteman, Jesus Gonzalez Bosquet, Daniel Petereit, Diane Harper, Sarah H. Nash

**Affiliations:** ^1^ Department of Obstetrics and Gynecology, Division of Gynecologic Oncology, University of Iowa Hospitals and Clinics, Iowa City, Iowa, USA.; ^2^ Iowa Cancer Registry, Department of Epidemiology, College of Public Health, University of Iowa, Iowa City, Iowa, USA.; ^3^ Holden Comprehensive Cancer Center, University of Iowa, Iowa City, Iowa, USA.; ^4^ Monument Health, Rapid City, South Dakota, USA.; ^5^ Department of Obstetrics and Gynecology, University of Michigan, Ann Arbor, Michigan, USA.

**Keywords:** native American, cervical cancer, ovarian cancer, gynecologic oncology, health disparities, cancer equity

## Abstract

**Background:**

American Indian and Alaska Native (AI/AN) women experience higher rates of mortality from many cancers than their non-Native counterparts.

**Objective:**

To examine recent data on gynecological cancers (cervical, ovarian, and uterine) among AI/AN women living in the Upper Midwest (Iowa, Montana, Nebraska, North Dakota, South Dakota, and Wyoming) for any improvement in equity.

**Methods:**

We used data from the North American Association for Central Cancer Registries Cancer in North America database (1995–2019). We used descriptive statistics, including incidence mortality rates, trends, and time to treatment. Analyses were restricted to non-Hispanic individuals living in a purchased/referred care delivery area (PRCDA) at the time of diagnosis; sensitivity analyses included all AI/AN people, regardless of PRCDA residence or ethnicity.

**Results:**

From 1995 to 2019, there were 647 gynecological cancers diagnosed among AI/AN women living in PRCDA counties in the Upper Midwest (cervical *n* = 194, ovarian *n* = 142, uterine *n* = 311). Incidence and mortality rates for ovarian and uterine cancers were similar between AI/AN and non-Hispanic White (NHW) women; however, the incidence of cervical cancer was 1.87 (95% confidence interval [CI]: 1.60, 2.17) times higher, and mortality was 2.92 (95% CI: 2.29, 3.68) times higher among AI/AN compared to NHW women. The majority of AI/AN women diagnosed with gynecological cancer initiated treatment within 1 month (cervical = 67.2%, ovarian = 80.6%, uterine = 63.1%), which was similar to NHW women.

**Conclusions:**

Differences exist in incidence and mortality for cervical cancer between AI/AN and NHW women in the Upper Midwest, with AI/AN facing continued inequity.

## Introduction 

American Indian and Alaska Native (AI/AN) people face significant structural and social inequities that drive downstream cancer disparities, such as higher cancer incidence and mortality,^1–4^ and lower cancer survival.^4–6^ For example, a higher proportion of AI/AN people live in poverty and face unemployment compared to non-Hispanic Whites(NHW). The median income is almost half among AI/AN people as it is among NHW.^7^ Further, 27% of AI/AN people are uninsured, compared to 10% of U.S. Whites (Hispanic and non-Hispanic; United States White).^6^ In part due to forced displacement and relocation required by the U.S. government,^8–10^ many AI/AN people (approximately two-thirds) live in tribal reservations or surrounding counties. Healthcare in these counties, known as purchased/referred care delivery areas (PRCDAs), is provided by the Indian Health Service (IHS), a government agency directed by congressional mandate to provide healthcare to AI/AN people.^6^ The IHS has been consistently underfunded.^11^ For example, of the $48 billion per year required to fund IHS fully, only 13% ($6.23 billion) was budgeted for fiscal year 2021.^4^ This chronic underfunding has impacted healthcare access and quality for AI/AN people. Many specialty care services, including cancer care, are unavailable in IHS,^12,13^ or referrals to specialty care may be delayed.^11^ The result is that many AI/AN people receive lower-quality cancer care than their NHW counterparts.^14,15^

Access to specialty care is essential for the treatment of gynecological cancers.^16,17^ There are known disparities in the incidence of and mortality from gynecological malignancies among AI/AN women. AI/AN women living in PRCDA counties experience, on average, a 56% higher incidence of cervical cancer compared to their NHW counterparts,^4^ with disparities differing by region. Cervical cancer incidence rates are almost twice as high among Northern Plains and Pacific Northwest AI/AN women and 1.6 times higher among Alaska and Southern Plains AI/AN women compared to NHW.^1,18^ AI/AN women are also more likely to be diagnosed with cervical cancer at a later stage than NHW.^3,19^ Further, in 2019, ovarian cancer incidence was highest among AI/AN compared to all other racial/ethnic groups, at 11.4/100,000.^20^ While the research on access to gynecological care among AI/AN women is sparse, one study showed that 23.7% of AI/AN women did not have access to a physician within 100 miles between 2016 and 2020, which did not improve over time.^21^ Differences in socioeconomic factors, access to healthcare, and availability of specialty cancer care likely to contribute to regional variations in disparities faced by AI/AN with gynecological cancer in the United States.

Our study sought to examine the incidence and mortality of AI/AN women from gynecological cancer among those living in the Upper Midwest area (MT, WY, ND, SD, NE, and IA). These states are more formally designated as the Great Plains and Billings by the IHS. We focused specifically on this area due to the high burden of cervical cancer among AI/AN women living in the Northern Plains,^4,22,23^ the lack of published data regarding ovarian and uterine cancer in this area, and the lack of practicing gynecological oncologists in this region. According to the Society for Gynecologic Oncology, only seven cities in these states currently have licensed gynecological oncologists, including Billings, MT; Rapid City, SD; Sioux Falls, SD; Fargo, ND; Omaha, NE; Des Moines, IA; and Iowa City, IA. Our central hypothesis was that AI/AN women in the Upper Midwest were likely to have a high burden of gynecological cancers, particularly when compared to NHW women in the same region.

## Methods

### Ethics and data availability

The Institutional Review Board at the University of Iowa considered this study exempt. North American Association of Central Cancer Registries (NAACCR) data are available to qualified researchers through an application at naaccr.org.

### Study population

The 2020 Census indicated that there are currently 7.3 million American Indian/Alaska Native people (AI/AN; alone or in combination) in the United States.^24^ There are 574 federally recognized tribes and 324 reservations within the United States,^25^ with the majority of reservations located in rural locations. In Iowa, Montana, Nebraska, North Dakota, South Dakota, and Wyoming, there are 29 federally recognized tribes and/or tribal reservations. Approximately 290,000 AI/AN people live in this area of ∼525,000 sq miles.^26^

This study examined AI/AN and NHW residing in PRCDAs counties in the states above. These states were chosen due to several factors, including the largely rural population of both AI/AN and NHW, the density of reservations in this area, and the lack of access to specialty care, specifically gynecological oncologists.

### Data source

Retrospective incidence data for patients diagnosed with cervical, uterine, and ovarian cancers diagnosed between 1995 and 2019 in select Upper Midwest states were obtained from the NAACCR Cancer in North America (CiNA) database. The CiNA database includes cancer incidence data for all U.S. states and Washington DC, as well as Canadian provinces, from 1995 to the most recent year of data submission (at the time of our data request, 2019).^27^ The database includes data from both the Surveillance, Epidemiology, and End Results (SEER) registries and the National Program of Cancer Registries (NPCR). Patients eligible for inclusion in this primary analysis were those diagnosed with primary cervical, uterine, and ovarian cancer (ICD-O-3 codes C54.0, C54.1, C54.2, C54.3, C54.8, C54.9, C55.9, C53.0, C53.1, C53.8, C53.9, and C56.9), of American Indian/Alaska Native or White race, of non-Hispanic ethnicity, living in PRCDA counties in selected states (Iowa, Montana, Nebraska, North Dakota, South Dakota, or Wyoming). In a supplementary analysis, we included all AI/AN, regardless of PRCDA residence or ethnicity. Further, we included only natal sex females and patients aged 20 years or older at diagnosis (*i.e.,* nonpediatric cancer cases). Mortality data were from the National Center for Health Statistics, available through SEER*Stat.^28^

### Measures

Demographic variables collected included age at diagnosis, health insurance coverage at diagnosis, NAACCR registry (*i.e.,* state), rurality, and year of diagnosis. Age at diagnosis was categorized as 20–39, 40–59, and 65+ years. Health insurance coverage at diagnosis was categorized as private, Medicare, Medicaid/VA/TRICARE/Military, Indian/Public Health Service, and uninsured. The NAACCR registry variable included the states of Iowa, Montana, Nebraska, North Dakota, South Dakota, and Wyoming. Rural–urban continuum codes were used to measure rurality, with codes 1–3 categorized as urban and 4–9 as rural. The years of diagnosis were categorized into five categories (1995–1999, 2000–2004, 2005–2009, 2010–2014, 2015–2019).

Tumor characteristics include site (cervical, uterine, and ovarian), SEER summary stage at diagnosis (localized, regional, and distant), month and year of diagnosis, month and year of first-course therapy, and information on first-course treatment regimen (surgery, chemotherapy, radiation, immunotherapy, and hormone therapy). A crosswalk between SEER summary stage and International Federation of Gynecology and Obstetrics stage for each site is listed in Supplementary Table S1.

### Statistical analysis

We calculated descriptive statistics for demographic and clinical variables among AI/AN and NHW groups stratified by cancer site. We examined differences between AI/AN and NHW women for each cancer site using chi-squared tests or Fisher’s exact test where cell sizes were less than five. Incidence and mortality rates with 95% confidence intervals were calculated for each cancer site and race group in SEER*Stat and age-adjusted to the 2000 Standard U.S. Population. These analyses compared AI/AN women to NHW women. The choice of NHW as a comparison group was made (a) because NHW is the largest racial/ethnic group, leading to the most statistically stable comparisons, and (b) to maintain consistency with the large body of disparities literature that compares AI/AN people to NHW.^2,3,29,30^ However, we recognize that this choice does not necessarily align with anti-colonial research approaches.^31,32^

We also described and examined the time from diagnosis to cancer treatment for each cancer site and assessed differences by race, age at diagnosis, and stage at diagnosis. We calculated the time from the month/year of diagnosis to the month/year of first treatment. Since exact date information was unavailable in the NAACCR dataset, the day of diagnosis and treatment was set as the first of the respective month for all cases. To illustrate when patients were receiving cancer treatments, we created figures showing time to treatment categorized as less than 1 month, 2 months, 3 months, and 4 or more months. Differences in the meantime to treatment between groups were determined using ANOVA.

Primary analyses were restricted to PRCDA counties, as AI/AN racial misclassification is known to be lower in these counties.^33^ All NAACCR registries are linked to the IHS to reduce racial misclassification.^33^ Further, because of concerns with overestimates of Hispanic AI/AN in the Census population data, primary analyses were also restricted to non-Hispanic AI/AN (hereafter referred to as AI/AN for brevity). We conducted sensitivity analyses including all AI/AN (including non-PRCDA and Hispanic AI/AN) for incidence and mortality rates and rate ratios. All analyses were performed in SAS (SAS 9.4, SAS Institute, Cary, NC). A *p* value of <0.05 was considered statistically significant.

## Results

We examined demographic and clinical characteristics for gynecological cancers (cervical, ovarian, and uterine) diagnosed in Iowa, Montana, Nebraska, North Dakota, South Dakota, and Wyoming between 1995 and 2019, stratified by race (AI/AN, NHW) and cancer site in Table 1. For all cancer sites, a smaller proportion of AI/AN women were diagnosed after the age of 60 years (cervical *p* = 0.10, ovarian *p* < 0.0001, uterine *p* < 0.0001) relative to NHW. There were also differences in stage at the time of diagnosis between AI/AN and NHW women. For cervical cancers, AI/AN women had a slightly lower proportion diagnosed at the local stage and a higher proportion at the regional stage (*p* = 0.10). For ovarian cancers, AI/AN women had a higher proportion diagnosed at local stage (*p* = 0.03). AI/AN were also less likely to have private insurance or Medicare for all disease sites.

**Table 1.** Characteristics of 1995–2019 Cervical, Ovarian, and Uterine Cancer Patients in Iowa, Montana, Nebraska, North Dakota, South Dakota, and Wyoming, Stratified by Race and Cancer Site

	Cervical			Ovarian			Uterine		
	*n* (col%)	*n* (col%)	*p*	*n* (col%)	*n* (col%)	*p*	*n* (col%)	*n* (col%)	*p*
	AI/AN (*n* = 194)	NHW (*n* = 1802)		AI/AN (*n* = 142)	NHW (*n* = 3600)		AI/AN (*n* = 311)	NHW (*n* = 8058)	
NAACCR Registry			<0.0001			<0.0001			<0.0001
Iowa	^a^	212 (12)		^a^	387 (11)		^a^	807 (10)	
Montana	55 (28)	366 (20)		49 (35)	836 (23)		119 (38)	1639 (20)	
Nebraska	^a^	890 (49)		13 (9)	1583 (44)		19 (6)	3721 (46)	
North Dakota	35 (18)	106 (6)		22 (15)	300 (8)		43 (14)	669 (8)	
South Dakota	80 (41)	153 (8)		41 (29)	338 (9)		103 (33)	974 (12)	
Wyoming	^a^	75 (4)		13 (9)	157 (4)		19 (6)	248 (3)	
Age at diagnosis			0.10			<0.0001			<0.0001
20–39 years	63 (32)	507 (28)		13 (9)	171 (5)		37 (12)	208 (3)	
40–59 years	90 (46)	786 (44)		67 (47)	1131 (31)		158 (51)	2659 (33)	
60+ years	41 (21)	509 (28)		62 (44)	2297 (64)		116 (37)	5191 (64)	
Year of diagnosis			0.0008			0.05			0.002
1995–1999	16 (8)	353 (20)		14 (10)	619 (17)		22 (7)	1128 (14)	
2000–2004	34 (18)	359 (20)		28 (20)	785 (22)		50 (16)	1484 (18)	
2005–2009	42 (22)	341 (19)		32 (23)	807 (22)		79 (25)	1644 (20)	
2010–2014	52 (27)	355 (20)		30 (21)	717 (20)		74 (24)	1884 (23)	
2015–2019	50 (26)	394 (21)		38 (27)	672 (19)		86 (28)	1918 (24)	
Health insurance coverage			<0.0001			<0.0001			<0.0001
Private	12 (7)	490 (35)		13 (11)	737 (26)		31 (11)	1852 (28)	
Medicare	14 (8)	280 (20)		31 (26)	1334 (46)		73 (27)	2980 (45)	
Medicaid	81 (48)	511 (36)		36 (30)	621 (22)		70 (25)	1494 (23)	
Indian/Public Health Service, TRICARE, Veterans Affairs	54 (32)	0 (0)		34 (29)	0 (0)		90 (33)	0 (0)	
Uninsured	^a^	128 (9)		^a^	160 (6)		11 (4)	299 (5)	
Missing	25	393		23	748		36	1431	
Stage at diagnosis			0.10			0.03			0.11
Local	80 (46)	911 (54)		34 (27)	574 (18)		213 (74)	5746 (75)	
Regional	72 (41)	567 (33)		21 (17)	636 (20)		43 (15)	1306 (17)	
Distant	23 (13)	222 (13)		71 (56)	2035 (63)		32 (11)	602 (8)	

^a^ Cell sizes less than 10 have been censored.

AI/AN, American Indian and Alaska Native; NAACCR, North American Association of Central Cancer Registries; NHW, non-Hispanic Whites.

When comparing AI/AN and NHW incidence and mortality rates by state (Table 2), we only observed significant differences for cervical cancers, where the incidence rate ratio was 1.87 (1.61, 2.17) and the mortality rate ratio was 2.69 (2.17, 3.31). No differences in rates existed for other cancer sites. Sensitivity analyses, including rates for non-PRCDA and Hispanic AI/AN women, are presented in Supplementary Table S1. Incidence and mortality rates for each cancer site varied by state. For example, cervical cancer incidence ranged from 8.5/100,000 (4.8, 14.2) in Nebraska to 17.9/100,000 (14.1, 22.4) in South Dakota. Incidence of uterine cancer varied from 13.9/100,000 (8.0, 22.4) in Nebraska to 28.0/100,000 (23.3, 33.2) in Montana. Across sites and states, rates were generally higher when all AI/AN were included, regardless of PRCDA residence or Hispanic ethnicity.

**Table 2.** Incidence and Mortality Rates, 95% CI, and Rate Ratios for 1995–2019 Cervical, Ovarian, and Uterine Cancer Patients in Iowa, Montana, Nebraska, North Dakota, South Dakota, and Wyoming, Stratified by Race and Cancer Site

	Cervical				Ovarian				Uterine				
	AI/AN		NHW		AI/AN		NHW		AI/AN		NHW		
	IR (95% CI)	MR (95%CI)	IR (95% CI)	MR (95% CI)	IR (95% CI)	MR (95% CI)	IR (95% CI)	MR (95% CI)	IR (95% CI)	MR (95% CI)	IR (95% CI)	MR (95% CI)	
Iowa	12.4 (4.5, 28.3)	^b^	8.8 (7.7, 10.0)	2.9 (2.7, 3.1)	12.6 (3.8, 30.5)	^b^	12.1 (10.9, 13.4)	11.9 (11.5, 12.2)	22.1 (10.0, 42.5)	^b^	25.4 (23.7, 27.2)	6.7 (6.4, 7.0)	
Montana	10.7 (8.1, 13.8)	6.2 (4.0, 9.2)	6.7(6.0, 7.4)	2.5 (2.1, 2.8)	12.6 (9.5, 16.4)	9.1 (6.2, 12.8)	12.7 (11.8, 13.6)	11.8 (11.2, 12.5)	28.0 (23.3, 33.2)	6.3 (3.8, 9.9)	24.1 (23.0, 25.4)	6.0 (5.5, 6.5)	
Nebraska	8.5 (4.8, 14.2)	6.5 (3.1, 12.7)	7.6 (7.2, 8.1)	3.0 (2.7, 3.3)	14.1 (7.0, 24.5)	16.6 (8.4, 28.6)	11.6 (11.0, 12.2)	10.7 (10.2, 11.2)	13.9 (8.0, 22.4)	^b^	26.8 (25.9, 27.6)	6.5 (6.2, 6.9)	
North Dakota	13.9 (9.5, 19.6)	5.7 (2.9, 10.2)	5.2 (4.2, 6.3)	2.3 (2.0, 2.7)	10.2 (6.4, 15.3)	10.4 (6.0, 16.6)	11.4 (10.1, 12.8)	10.2 (9.5, 11.0)	18.8 (13.5, 25.3)	^b^	25.1 (23.2, 27.2)	5.6 (5.1, 6.2)	
South Dakota	17.9 (14.1, 22.4)	11.1 (8.1, 14.8)	5.9 (5.0, 7.0)	2.0 (1.7, 2.4)	10.3 (7.3, 14.0)	9.1 (6.2, 12.9)	10.0 (9.0, 11.2)	11.5 (10.8, 12.3)	24.2 (19.7, 29.4)	5.7 (3.4, 8.9)	28.5 (26.7, 30.4)	5.9 (5.4, 6.4)	
Wyoming	14.3 (6.8, 26.0)	^b^	7.2 (5.6, 9.1)	3.2 (2.7, 3.8)	21.9 (12.1, 35.9)	13.3 (6.2, 24.6)	12.3 (10.4, 14.4)	11.6 (10.6, 12.5)	25.5 (15.2, 39.9)	^b^	18.3 (16.1, 20.8)	5.3 (4.6, 5.9)	
All States	13.5 (11.6, 15.5)	7.4 (6.0, 9.1)	7.2 (6.9, 7.5)	2.8 (2.6, 2.9)	12.1 (10.2, 14.2)	9.8 (8.0, 11.9)	11.7 (11.4, 12.1)	11.4 (11.2, 11.6)	23.7 (21.1, 26.5)	5.1 (3.8, 6.8)	25.7 (25.2, 26.3)	6.3 (6.1, 6.5)	
Rate ratio and 95% CI^a^	1.87 (1.61, 2.17)	2.69 (2.17, 3.31)	REF	REF	1.03 (0.87, 1.21)	0.86 (0.70, 1.05)	REF	REF	0.92 (0.82, 1.03)	0.82 (0.60, 1.07)	REF	REF	

^a^ Rate ratios are calculated comparing AI/AN to NHW by cancer site.

^b^ Statistics not displayed due to fewer than 10 cases.

Restricted to individuals living in purchased/referred care delivery area (PRCDA) counties and of non-Hispanic ethnicity. Incidence and mortality rates are per 100,000 and age-adjusted to the 2000 U.S. Standard Population (19 age groups—Census P25-1130).

CI, confidence interval; IR, incidence rate; MR, mortality rate.

**FIG. 1.**
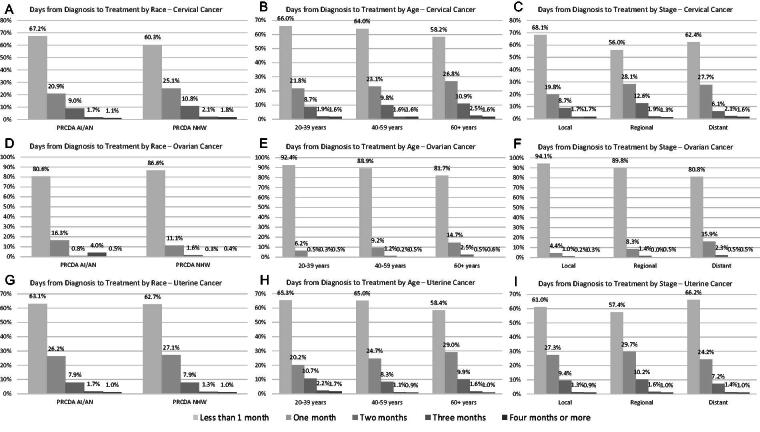
Mean time to treatment,* 1995–2019 cervical, ovarian, and uterine cancer patients in Iowa, Montana, Nebraska, North Dakota, South Dakota, and Wyoming, stratified by race and cancer site. **(A)** Cervical cancer by race, **(B)** cervical cancer by age, **(C)** cervical cancer by stage, **(D)** ovarian cancer by race, **(E)** ovarian cancer by age, **(F)** ovarian cancer by stage, **(G)** uterine cancer by race, **(H)** uterine cancer by age, and **(I)** uterine cancer by stage. *Since exact date information was unavailable in the NAACCR dataset, the day of diagnosis and treatment was set as the first of the respective month for all cases. NAACCR, North American Association of Central Cancer Registries.

Most patients received cancer treatment within 2 months of diagnosis (Fig. 1). There were no differences in the distribution of treatment times between AI/AN and NHW for cervical (*p* = 0.22), ovarian (*p* = 0.12), or uterine (*p* = 0.13) cancers. When examined by age, we found that for all cancer sites, the mean time to treatment increased with age at diagnosis (all *p* < 0.01). The pattern by stage differed by cancer site, with regional stage having the highest mean time to treatment for cervical and uterine cancer (*p* < 0.001), and the distant stage having the highest mean time to treatment for ovarian cancer (*p* < 0.001). Time to treatment also varied by state of residence (all *p* < 0.001). The data supporting Figure 1 are presented in Supplementary Table S2.

## Discussion

In this sample of AI/AN women living in the Upper Midwest diagnosed with cervical, uterine, and ovarian cancers between 1995 and 2019, our primary finding was that incidence of and mortality from cervical cancer remains disproportionately high compared to NHW. AI/AN women were also more likely than NHW to be diagnosed with regional or distant stage cervical cancer. We observed no differences in the incidence of or mortality from ovarian or uterine cancers between AI/AN and NHW. However, AI/AN women were more likely than NHW to be diagnosed with regional or distant uterine cancers and more likely to be diagnosed with local ovarian cancer. We were also interested in how quickly AI/AN women were able to access treatment, given the geographic isolation and lack of access to specialty care, especially for those living on the tribal reservations. We saw that most women, AI/AN and NHW, initiated treatment within 2 months of their diagnosis and that there were no differences in time to treatment between AI/AN and NHW women across all cancer types. Together, our findings indicate that while there remain disparities in incidence, mortality, and stage at diagnosis, particularly for cervical cancer, time to treatment does not seem to be a primary contributor to differences in outcome for gynecological malignancies for AI/AN women in this area.

Our observation that cervical cancer incidence and mortality were higher among AI/AN than NHW women living in the Upper Midwest is in line with existing literature examining cervical cancer incidence and mortality disparities nationally.^1,4,34^ Indeed, AI/AN have the second highest cervical cancer mortality rates among all racial and ethnic groups, behind Hispanic individuals.^35^ Higher mortality rates may, at least in part, be driven by a later stage at diagnosis. AI/AN women in our study were more likely to be diagnosed with regional/distant stage disease and less likely to be diagnosed with local stage disease than NHW. This finding concurs with national data,^1^ as well as a study focused on AI/AN women in the Pacific Northwest.^18^ It is of great concern that although these disparities have been well described over time, the burden of cervical cancer among AI/AN in the Upper Midwest and the larger Northern Plains area remains persistent over time. Further, while there is significant cultural diversity across AI/AN populations, it is troubling that cervical cancer disparities appear to be constant. A recent review highlighted the lack of research contributing to our understanding of these disparities.^36^

Several factors may explain the persistently elevated burden of cervical cancer among AI/AN women, particularly those living in the Upper Midwest. This includes increased prevalence of high-risk, persistent human papillomavirus (HPV) infection, lower prevalence of screening and HPV vaccination, possible decreased follow-up of abnormal results, and the provision of healthcare to AI/AN. In the Northern Plains, HPV infection prevalence is higher than in the general U.S. population, with 35% of AI/AN people testing positive for at least one high-risk HPV type.^37^ In a study conducted among rural South Dakotan AI/AN women and urban NHW women, the AI/AN women were more likely to present with HPV infections that were not preventable *via* HPV vaccination (32% [95% confidence interval (CI) = 26–38] vs. 15% [95% CI = 11–21]).^38^ Lower use of preventive measures may also contribute. In the Upper Midwest, screening prevalence is low, most recently reported at 46%.^4^ Another possible contributor is follow-up of abnormal screening tests. In areas such as the Upper Midwest that have large land areas and relatively few specialty providers, this could present a barrier to care, influencing the increased incidence/mortality among AI/AN women. As there are notable differences in the pathophysiology of HPV infection, screening rates, and socioeconomic and geographic differences in the Upper Midwest/Plains, further dedicated research specific to this geographic area is sorely needed to find actionable and realistic interventions to increase and optimize cervical cancer screening.

One challenge to decreasing known cervical cancer disparities among AI/AN women across the United States is the provision of healthcare to this population. Most AI/AN people receive care under the government-operated IHS, which provides healthcare services for enrolled tribal citizens through clinics and hospitals.^39^ Approximately 1/3 of IHS patients are uninsured, which makes them completely reliant on IHS.^4^ Our results reflected this, which demonstrated AI was less likely to have private insurance or Medicare. IHS is consistently underfunded, spending approximately 40% less per capita than Veterans Affairs.^39^ As a result, many facilities do not offer cancer screening, and full coverage of cancer prevention services is not guaranteed to IHS or tribal healthcare beneficiaries.^40,41^ Accessing specialty cancer care through IHS can be particularly challenging and often requires referral to out-of-system providers. For example, there are only 20 practicing gynecological oncologists in the Upper Midwest, none of whom work for the IHS. Access to care can also be a challenge for patients, who may require transportation or temporary relocation for treatment at geographically distant facilities,^35^ which in turn may lead to delays in time to treatment and/or completion of treatment.^41–43^ Designating referral centers may be one strategy to streamline the transfer of care from IHS facilities to higher-volume hospitals with the required specialty care.^35^ Given that high-quality specialist care by gynecological oncologists results in improved outcomes, ensuring access to these services for AI/AN women will be paramount in reducing those disparities observed herein and elsewhere.

Despite numerous challenges, there have been some successes in tribally and IHS-led initiatives to improve access to cervical cancer prevention. For example, a recent study examined best practices for HPV vaccination at IHS, tribally operated, and Urban Indian Healthcare facilities. It implemented feasible interventions for facilities failing to reach national targets.^44^ Evidence-based strategies implemented to increase vaccination coverage included standing orders for vaccinations, providing vaccinations at nurse-only visits, and patient/parent education. Efforts to address HPV vaccination among AI/AN people have shown success, with a higher proportion of AI/AN youth having received at least one dose of the HPV vaccination (85% vs. 71%) or having completed the vaccination series (66% vs. 55%), compared to NHW.^45^ Further, the National Breast and Cervical Cancer Detection Program (NBCCDP) was formally extended to AI/AN women in 1993 through partnerships with tribal organizations.^46^ The success of the NBCCDP led to progressive increases in the total no. of AI/AN women screened. Unfortunately, in the Upper Midwest, only two Tribal NBCCDP programs exist, which leads to a vast area with a high density of AI/AN people being underserved by this program.

Another possible solution is community-based interventions. One example of this is the Walking Forward Program, which is an National Cancer Institute (NCI)-funded program in South Dakota. This program works with and functions in AI/AN communities to facilitate cancer screening events, follow-up of abnormal results, and expedite referrals for cancer treatment. Increasing patient navigation and the longevity of patient advocates and providers has shown great success in this area, especially among AI/ANs who often distrust the medical system due to their experiences with IHS.^47^ Continued focus and funding should be given to support existing efforts and grow the capacity of tribal and community organizations to implement culturally appropriate, evidence-based preventive interventions. Further, community-specific considerations, such as testing and vaccination that better reflect HPV strains observed, may be warranted.

Our study found no difference between AI/AN and NHW in ovarian and uterine cancer incidence and mortality. However, AI/AN women were more likely to be diagnosed with local-stage ovarian cancer and with regional or distant stage uterine cancers. Our findings are notable because of the lack of data published for AI/AN populations in this area for these gynecological cancer types. Further, while the literature is limited and there are mixed results regarding a disparity (or lack thereof), many studies fail to detect statistically significant differences despite elevated rates due to small sample sizes and wide confidence intervals.^48,49^ One consideration for these malignancies may be the lower life expectancy among AI/AN women. The average life expectancy of AI/AN is 65.2 years, the lowest of any racial or ethnic group in the United States.^50^ As the average age at diagnosis is 60 years for uterine cancer and 63 years for ovarian cancer, competing risks may be a factor in our study and others before finding no significant differences in incidence and mortality from these cancers.^51,52^ Further, since some risk factors for uterine/endometrial cancers (*e.g.,* obesity, diabetes) are higher among AI/AN people compared to NHW, continued monitoring of these trends may be necessary.

This study has several strengths and limitations that warrant consideration in interpreting its findings. First, we used data from high-quality cancer registries funded by the SEER and NPCR, available from the NAACCR, for this analysis. This enabled us to look at cancer incidence and mortality among AI/AN women residing in states across the Midwest, which is novel. Misclassification of AI/AN race in cancer registries is known.^33^ However, all states routinely link to IHS records, which increases accuracy, particularly in PRCDA counties. We limited our analyses to PRCDA counties and non-Hispanic AI/AN people to reduce the potential for differential racial misclassification by state. We observed wide confidence intervals for some of our analyses, which may have impacted our ability to observe statistically significant differences between groups. However, despite its challenges, the importance of small-population cancer research has been well described.^53^ Finally, precise dates of treatment were unavailable for our time-to-treatment analyses. To account for this, we assigned each individual a diagnosis and treatment date of the 15th of the month. This likely resulted in misclassification, leading to overestimating the time to care. However, any misclassification would have been non-differential and would not have affected comparisons between groups.

In conclusion, our study emphasized that despite improved efforts for HPV vaccination and screening programs, cervical cancer incidence and mortality remain an ongoing disparity affecting AI/AN women in the Upper Midwest. As there are many differences between geographic regions regarding HPV strains, screening rates, and other societal factors, further research is needed specific to this area to explore this ongoing disparity further. In contrast to some other studies, we did not observe any disparities in ovarian and uterine cancers in this study. Further monitoring and evaluation may be necessary as risk factors for the development of endometrial cancer are higher among the AI/AN population (*e.g.,* diabetes, obesity). Interestingly, we did not observe differences in time to treatment between AI/AN and NHW women. However, known barriers to access to care may affect both populations equally. Given the low amount of gynecological oncology specialists in the region, further research should determine whether observed differences in mortality and survival across gynecological malignancies are due to late diagnoses or other factors related to the quality of treatment beyond timeliness.

## Abbreviations Used

AI/AN: American Indian/Alaska Native
CiNA: Cancer in North America
FIGO: International Federation of Gynecology and Obstetrics
HPV: Human Papillomavirus
IHS: Indian Health Service
NAACR: North American Association for Central Cancer Registries
NBCCDP: National Breast and Cervical Cancer Detection Program
NCI: National Cancer Institute
NHW: Non-Hispanic White
PRCDA: Purchased/Referred Care Delivery Area
SEER: Surveillance, Epidemiology, and End Results
USW: United States White
