# Supplementary material for: Gynecological Cancers Among American Indian and Alaska Native Women Living in the Upper Midwest, 1995–2019
Source: Womens Health Rep (New Rochelle). 2025 Feb 25;6(1):199–208. doi: 10.1089/whr.2024.0124 (PMC11932641; doi:10.1089/whr.2024.0124)
Supplement: Supplementary Table S1 [file whr.2024.0124_supplementary_table_s1.docx]

**Supplementary Table 1. NAACCR registry/SEER registry summary stage and corresponding FIGO staging**

| Cancer Type | Registry Definition | Corresponding FIGO Staging |
| --- | --- | --- |
| Cervical | Local | Stage I (IA1, IA2, IA NOS, IB1, IB2, IB3, IB NOS, I NOS) |
|  | Regional | Stage II (IIA, IIB, II NOS)  Stage III (IIIA, IIIB, IIIC1, IIIC2, IIIC NOS, III NOS) |
|  | Distant | Stage IV (IVA, IVB, IV NOS) |
| Uterine | Local | Stage I (IA, IB, I NOS) |
|  | Regional | Stage II  Stage III (IIIA, IIIB, IIIC1, IIIC2, IIIC NOS, III NOS) |
|  | Distant | Stage IV (IVA, IVB, IV NOS) |
| Ovarian | Local | Stage I (IA, IB, IC1, I NOS) |
|  | Regional | Stage IC2, IC3 Stage II (IIA, IIB, II NOS)  Stage IIIA1 (IIA1i, IIIA1ii, IIIA1 NOS) |
|  | Distant | Stage III (IIIA, IIIA2, IIIB, IIIC, III NOS)  Stage IV (IVA, IVB, IV NOS) |
