# Supplementary material for: Gynecological Cancers Among American Indian and Alaska Native Women Living in the Upper Midwest, 1995–2019
Source: Womens Health Rep (New Rochelle). 2025 Feb 25;6(1):199–208. doi: 10.1089/whr.2024.0124 (PMC11932641; doi:10.1089/whr.2024.0124)
Supplement: Supplementary Table S2 [file whr.2024.0124_supplementary_table_s2.docx]

**Supplementary Table 2.** Incidence and Mortality Rates, 95% CI, and Rate Ratios for 1995-2019 Cervical, Ovarian, and Uterine Cancer Patients in Iowa, Montana, Nebraska, North Dakota, South Dakota, and Wyoming, Stratified by Race and Cancer Site. Including all AI/AIN people, regardless of PRCDA residence or Hispanic ethnicity status.

Incidence and mortality rates are per 100,000 and age-adjusted to the 2000 US Std Population (19 age groups – Census P25-1130)

* Rate ratios are calculated comparing AI/AN to NHW by cancer site

^ Statistics not displayed due to fewer than 10 cases

|  | Cervical | | | | Ovarian | | | | Uterine | | | | |  |
| --- | --- | --- | --- | --- | --- | --- | --- | --- | --- | --- | --- | --- | --- | --- |
|  | AI/AN | | White | | AI/AN | | White | | AI/AN | | White | | |  |
|  | IR & 95%CI | MR & 95%CI | IR & 95%CI | MR & 95%CI | IR & 95%CI | MR & 95%CI | IR & 95%CI | MR & 95%CI | IR & 95%CI | MR & 95%CI | IR & 95%CI | MR & 95%CI |  |  |
| Iowa | 15.1 (8.1, 26.4) | ^ | 10.5 (10.2, 11.0) | 2.9 (2.7, 3.1) | 12.0 (5.4, 23.3) | ^ | 41.6 (40.9, 42.3) | 11.9 (11.5, 12.2) | 22.1 (13.3, 35.2) | ^ | 17.8 (17.3, 18.3) | 6.7 (6.4, 7.0) |  |  |
|  |  |  |  |  |  |  |  |  |  |  |  |  |  | |
| Montana | 15.4 (12.0, 19.5) | 6.2 (4.0, 9.2) | 9.7 (9.0, 10.4) | 2.5 (2.1, 2.8) | 18.9 (14.7, 24.0) | 9.1 (6.2, 12.8) | 34.1 (33.0, 35.3) | 11.8 (11.2, 12.5) | 39.9 (33.9, 46.7) | 6.3 (3.8, 9.9) | 18.0 (17.1, 18.9) | 6.0 (5.5, 6.5) |  | |
|  |  |  |  |  |  |  |  |  |  |  |  |  |  | |
| Nebraska | 12.5 (7.7, 19.7) | 6.5 (3.1, 12.7) | 10.7 (10.2, 11.3) | 3.0 (2.7, 3.3) | 18.8 (10.3, 30.9) | 16.6 (8.4, 28.6) | 38.6 (37.7, 39.6) | 10.7 (10.2, 11.2) | 24.7 (16.2, 36.3) | ^ | 16.5 (15.9, 17.1) | 6.5 (6.2, 6.9) |  | |
|  |  |  |  |  |  |  |  |  |  |  |  |  |  | |
| North Dakota | 19.0 (13.6, 26.1) | 5.7 (2.9, 10.2) | 7.7 (7.0, 8.6) | 2.3 (2.0, 2.7) | 14.7 (9.7, 21.4) | 10.4 (6.0, 16.6) | 35.8 (34.3, 37.4) | 1.2 (9.5, 11.0) | 29.0 (22.0, 37.6) | ^ | 15.9 (14.9, 17.0) | 5.6 (5.1, 6.2) |  | |
|  |  |  |  |  |  |  |  |  |  |  |  |  |  | |
| South Dakota | 26.0 (20.9, 32.0) | 11.1 (8.1, 14.8) | 8.2 (7.4, 9.1) | 2.0 (1.7, 2.4) | 15.5 (11.3, 20.6) | 9.1 (6.2, 12.9) | 38.5 (36.9, 40.1) | 11.5 (10.8, 12.3) | 35.6 (29.5, 42.7) | 5.7 (3.4, 8.9) | 15.9 (14.9, 17.0) | 5.9 (5.4, 6.4) |  | |
|  |  |  |  |  |  |  |  |  |  |  |  |  |  | |
| Wyoming | 17.4 (9.5, 29.3) | ^ | 11.4 (10.4, 12.5) | 3.2 (2.7, 3.8) | 20.6 (11.4, 33.9) | 13.3 (6.2, 24.6) | 30.4 (28.9, 32.0) | 11.6 (10.6, 12.5) | 29.6 (19.0, 44.1) | ^ | 17.8 (16.7, 19.1) | 5.3 (4.6, 5.9) |  | |
|  |  |  |  |  |  |  |  |  |  |  |  |  |  | |
| All States | 18.7 (16.4, 21.2) | 7.4 (6.0, 9.1) | 10.2 (9.9, 10.4) | 2.8 (2.6, 2.9) | 17.0 (12.6, 19.6) | 9.8 (8.0, 11.9) | 17.2 (16.9, 17.6) | 11.4 (11.2, 11.6) | 33.7 (30.4, 37.2) | 5.1 (3.8, 6.8) | 17.2 (16.9, 17.6) | 6.3 (6.1, 6.5) |  | |
|  |  |  |  |  |  |  |  |  |  |  |  |  |  | |
| Rate Ratio & 95% CI* | 1.84 (1.61, 2.09) | 2.69 (2.17, 3.31) | REF | REF | 0.98 (0.84, 1.14) | 0.86 (0.70, 1.05) |  | REF | 0.87 (0.79, 0.97) | 0.82 (0.60, 1.07) | REF | REF |  | |
|  |  |  |  |  |  |  |  |  |  |  |  |  |  | |

CI: Confidence Interval, IR: Incidence Rate, MR: Mortality Rate
