# Supplementary material for: Gynecological Cancers Among American Indian and Alaska Native Women Living in the Upper Midwest, 1995–2019
Source: Womens Health Rep (New Rochelle). 2025 Feb 25;6(1):199–208. doi: 10.1089/whr.2024.0124 (PMC11932641; doi:10.1089/whr.2024.0124)
Supplement: Supplementary Table S3 [file whr.2024.0124_supplementary_table_s3.docx]

**Supplementary Table 3**. Mean time to treatment (TTT) in days among 1995-2019 Cervical, Ovarian, and Uterine Cancer Patients in Iowa, Montana, Nebraska, North Dakota, South Dakota, and Wyoming, Stratified by Cancer Site, Age at Diagnosis, and Race.

|  | Mean TTT (SD) | ANOVA p-value |
| --- | --- | --- |
| *Cervical* | | |
| NAACCR Registry |  | <0.001 |
| Iowa | 22.5 (28.8) |  |
| Montana | 26.0 (31.7) |  |
| Nebraska | 24.7 (31.9) |  |
| North Dakota | 20.8 (26.0) |  |
| South Dakota | 18.4 (22.3) |  |
| Wyoming | 20.8 (28.9) |  |
| Age at Diagnosis |  | <0.001 |
| 20-44 years | 20.8 (28.4) |  |
| 45-64 years | 23.7 (31.0) |  |
| 65+ years | 26.1 (27.9) |  |
| Race |  | 0.22 |
| AI/AN | 20.3 (26.1) |  |
| NHW | 23.0 (29.5) |  |
| Stage |  | <0.001 |
| Local | 20.3 (30.5) |  |
| Regional | 26.8 (27.2) |  |
| Distant | 22.8 (28.9) |  |
| *Ovarian* | | |
| NAACCR Registry |  | <0.001 |
| Iowa | 9.5 (18.5) |  |
| Montana | 9.5 (20.7) |  |
| Nebraska | 8.1 (20.2) |  |
| North Dakota | 10.4 (22.4) |  |
| South Dakota | 5.6 (13.0) |  |
| Wyoming | 9.0 (19.1) |  |
| Age at Diagnosis |  | <0.001 |
| 20-44 years | 4.2 (12.6) |  |
| 45-64 years | 7.6 (17.5) |  |
| 65+ years | 11.1 (21.4) |  |
| Race |  | 0.12 |
| AI/AN | 11.6 (21.5) |  |
| NHW | 8.9 (19.2) |  |
| Stage |  | <0.001 |
| Local | 3.7 (15.4) |  |
| Regional | 5.7 (16.6) |  |
| Distant | 11.2 (20.1) |  |
| *Uterine* | | |
| NAACCR Registry |  | <0.001 |
| Iowa | 24.9 (27.2) |  |
| Montana | 21.5 (25.1) |  |
| Nebraska | 22.7 (26.6) |  |
| North Dakota | 20.5 (23.7) |  |
| South Dakota | 19.0 (25.5) |  |
| Wyoming | 20.0 (25.3) |  |
| Age at Diagnosis |  | <0.001 |
| 20-44 years | 18.9 (28.1) |  |
| 45-64 years | 22.0 (25.8) |  |
| 65+ years | 24.3 (26.8) |  |
| Race |  | 0.13 |
| AI/AN | 20.6 (24.0) |  |
| NHW | 22.9 (26.5) |  |
| Stage |  | <0.001 |
| Local | 22.7 (26.2) |  |
| Regional | 24.8 (26.1) |  |
| Distant | 20.0 (25.0) |  |
